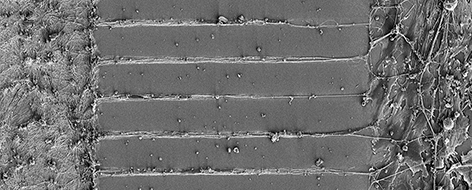# Searching for the primary site of motor neuron degeneration in ALS

**Published:** 2015-03

Amyotrophic lateral sclerosis (ALS) is a progressive, relentless neurodegenerative disease affecting upper and lower motor neurons. It is known that excitotoxicity plays a central role in ALS pathogenesis; however, the precise mechanism through which excitotoxicity develops and drives motor neuron degeneration, as well as the primary site (either the cell soma or axon) at which this pathological process is initiated, have still to be elucidated. To shed light on this, Tracey Dickson’s group used mouse spinal cord motor neurons and exposed them to site-specific excitotoxicity. The researchers found that these cells are vulnerable to somatodendritic but not axonal excitotoxin exposure. Similarly, excitotoxic insult to the cell body led to degeneration of neuromuscular junctions (the motor-neuron–muscle peripheral synapses) *in vivo*. These results suggest that excitotoxicity drives a die-forward mechanism of motor neuron death from the cell body outward to distal neuronal compartments. The model described could be used to test cell-soma-targeted interventions to treat ALS.

**Page 215**

**Figure:**